# Datasets on organizational citizenship behavior in the selected hospitals with different ownership

**DOI:** 10.1016/j.dib.2018.05.024

**Published:** 2018-05-19

**Authors:** Mohammad Ali Jahani, Shahrbanoo Mahmoudjanloo, Fatemeh Hoseini Rostami, Hosein Ali Nikbakht, Ghahraman Mahmoudi

**Affiliations:** a Social Determinants of Health Research Center, Health Research Institute,School of Medicine, Babol University of Medical Sciences, Babol, Islamic Republic of Iran; b Masters of Health Services Management, Mazandaran University of Medical Sciences, Sari, Islamic Republic of Iran; c Masters of Health Services Management, Golestan University of Medical Sciences, Gorgan, Islamic Republic of Iran; d Hospital Administration Research Center, School of Medicine, Sari Branch Islamic Azad University, Sari, Islamic Republic of Iran

**Keywords:** Hospitals, Ownership, Organizational citizenship behavior

## Abstract

Studying the role of employees as the base of an organization on achieving organizational goals has increased in recent years [1]. To have better organizational citizens, organizations should encourage their staff [2]. As the most powerful form of organizational behavior, organizational citizenship is more influential than organizational cooperation [3]. Studies have shown that cooperative behavior, such as the citizenship behavior results in easier organizational communication, promoting organizational planning, improving inter-personal cooperation and developing better organizational climate, directly influence staff satisfaction, work life quality, service-provision, job commitment and financial output [4]. As the most fundamental organizational behavior, the organizational citizenship behavior (OCB) causes so-called organizational effectiveness. This study Focused on the comparing organizational citizenship behavior components including conscientiousness, courtesy, altruism, sportsmanship and civic virtue among hospitals based on ownership. Research population included all therapeutic and non-therapeutic employees working in the five selected hospital located in Golestan province, Iran in 2016. This study is approved by Ethical committee of Islamic Azad Sari Branch. Based on Cochran׳s sampling formula, 312 employees working in different hospital sections and units (nurses and administrative personnel) were proportionally selected as the research sample. They completed the Persian version of Podsakoff׳s standard scale measuring organizational citizenship behavior. The data were analyzed using SPSS 22 and applying inferential statistics approaches such as t-test, Tukey, and ANOVA in the confidence interval of 95%.

**Specifications Table**

**Table:** 

Subject area	*Healthcare management*
More specific subject area	Comparing the organizational citizenship behavior in the selected hospitals with the kind of hospital ownership
Type of data	*Table, text file*
How data was acquired	– The Persian version of Podsakoff׳s standard scale for measuring organizational citizenship behavior was used for data collection. – After obtaining their consent, the subjects were asked to complete the scale by researchers׳ direct reference to the selected hospitals.
Data format	*Raw, analyzed, descriptive and statistical data*
Experimental factors	– Research population included all therapeutic and non-therapeutic employees working in the five selected hospital located in Golestan province, Iran in 2016. – Based on Cochran׳s sampling formulae, 312 employees working in different hospital sections and units (nurses and administrative personnel) were proportionally selected as the research sample.
Experimental features	Among the studied hospitals, the hospital owned by the social security had the highest mean rate of organizational citizenship behavior (see Table 1 for more details)
Data source location	*Golestan province, Iran*
Data accessibility	Data is included in this article.

**Value of the data**

• Data shows the relationship between organizational citizenship behavior and the kind of hospital ownership.
• Data compares the mean rates of the components of the organizational citizenship behavior (including conscientiousness, courtesy, altruism, sportsmanship and civic virtue) among the hospitals based on their kind of ownership.
• Findings showed a significant difference in the organizational citizenship behavior among the hospitals by their kind of ownership.

## 1 Data

Of 312 subjects, 241 (77.2%) were female employees. Most employees were in the age range of 30–39 years, 151 (48.4%). Most had BD degrees 251 (80.4%). Of them, 209 (67.0%) were therapeutic staff.

As shown in Table 1, the mean rate of women׳s organizational citizenship behavior (94.20 ± 8.98) was higher than that of men (93.83 ± 8.21). The mean rate of non- therapeutic/administrative employees׳ organizational citizenship behavior (94.38 ± 7.57) was higher than that of therapeutic employees (93.98 ± 9.36). Among the hospitals, the social security hospital significantly had the highest mean rate of organizational citizenship behavior (95.62 ± 7.20).

**Table 1:** The mean rate of employees׳ organizational citizenship behavior by their demographic information.

Variable	Groups	Frequency (%)	Mean ± SD	*P*-value
Gender	Men	71(22.8)	93.83 ± (8.21)	0.760
	Women	241(77.2)	94.20 ± (8.98)	
				
Age (year)	20–29	77(24.7)	93.82 ± (8.89)	0.451
	30–39	151(48.4)	93.83 ± (7.45)	
	40–49	68(21.8)	95.51 ± (8.99)	
	50≤	16(5.1)	92.31 ± (16.58)	
				
Educational degree	Public education	13(4.2)	91.46 ± (5.72)	0.457
	AD	31(9.9)	93.65 ± (7.90)	
	BD	251(80.4)	94.45 ± (9.21)	
	MD	17(5.4)	92.00 ± (4.97)	
				
Working length (year)	≤ 10	167(53.5)	93.93 ± (8.31)	0.695
	11–20	108(34.6)	94.65 ± (7.85)	
	21–30	37(11.9)	93.38 ± (12.86)	
				
Ownership	University Hospitals	258(82.7)	4.29 ± (8.46)	0.027
	Social security Hospital	34(10.9)	95.62 ± (7.20)	
	Private Hospital	20(6.4)	89.25 ± (8.80)	

As shown in Table 2, the mean rates of the components of the organizational citizenship behavior in the social security hospital were higher in the components conscientiousness (20.94 ± 2.59), courtesy (21.70 ± 1.86), and sportsmanship (19.00 ± 3.02) than those of other hospitals. In the components altruism (20.05 ± 1.36) and civic virtue (14.37 ± 2.20); however, the public hospitals had the higher rates.

**Table 2:** Mean ± SD of the components of organizational citizenship behavior based on the kind of hospital ownership in the studied hospitals.

Components	Conscientiousness	Courtesy	Altruism	Sportsmanship	Civic virtue
University hospitals	20.72 ± 2.42	20.70 ± 2.74	20.74 ± 2.67	17.74 ± 3.35	14.37 ± 2.20
Social security hospital	20.94 ± 2.59	21.70 ± 1.86	20.05 ± 1.36	19.00 ± 3.02	13.91 ± 1.99
Private hospital	19.20 ± 3.56	20.15 ± 3.46	20.50 ± 4.39	15.70 ± 2.67	13.70 ± 3.07

Table 3 shows the relationship between the components involved in the organizational citizenship behavior based on the kind of ownership in the studied hospitals. There was a significant relationship between the hospitals in the component "sportsmanship" (*p* = 0.05). The highest difference was in the relationship between private vs. other (University and social secure) hospitals. The differences in other components were not significant.

**Table 3:** Relationships between the components of organizational citizenship behavior based on the kind of hospital ownership in the studied hospitals.

Components	Kind of ownership	MD⁎	95% CI⁎	*p*-value⁎⁎	*p*-value⁎⁎
Conscientiousness	University -Social Security	−0.22	−1.30 - 0.86	0.882	0.028
	University -Private	1.52	0.13–2.90	0.027	
	Social Security-Private	1.74	0.06–3.41	0.040	
					
Curtsey	University -Social Security	0.99	−2.16-0.17	0.112	0.076
	University -Private	0.55	−0.92–2.04	0.649	
	Social Security-Private	1.55	−0.24–3.35	0.106	
					
Altruism	University -Social Security	0.68	−0.47–1.85	0.350	0.371
	University -Private	0.24	−1.23–1.72	0.920	
	Social Security-Private	−0.44	−2.24-1.35	0.832	
					
Sportsmanship	University -Social Security	−1.25	−2.66-0.15	0.091	0.002
	University -Private	2.04	0.24–3.83	0.021	
	Social Security-Private	3.30	1.12–5.47	0.001	
					
Civic virtue	University -Social Security	0.46	−0.49–1.42	0.494	0.258
	University -Private	0.67	−0.55–1.90	0.397	
	Social Security-Private	0.21	−1.27–1.70	0.940	

⁎ MD = Mean Difference, CI = Confidence.

⁎⁎ Significant levels for One-Way ANOVA and Tukey.

Table 4 shows the correlational matrix of the components involved in the organizational citizenship behavior in the studied hospitals. All components had significant relationship with each other (*p* < 0.01), except altruism-sportsmanship and sportsmanship-civic virtue.

**Table 4:** Correlational matrix of components involved in organizational citizenship behavior in the studied hospitals.

Components	Civic virtue	Sportsmanship	Altruism	Courtesy	Conscientiousness
Conscientiousness	0.361⁎	0.308⁎	0.316⁎	0.406⁎	1
Courtesy	0.239⁎	0.412⁎	0.430⁎	1	
Altruism	0.250⁎	0.026	1		
sportsmanship	0.020	1			
Civic virtue	1				

⁎ *p* < 0.001.

## 2 Experimental design, materials and methods

This applied study is a descriptive-analytical research conducted in 2016. Research population included all therapeutic and non-therapeutic employees working in the five selected hospital located in Golestan province, Iran (three university hospitals under supervision of Golestan University of Medical Science (Panj Azar Hospital, and Sayyad-e Shirazi Hospital located in Gorgan city and Al-jalil Hospital located in Agh-Ghala), Falsafi Private Hospital and Hakim-e Jorjani Hospital under supervision of Social Security Organization).

Based on Cochran׳s sampling formula, 312 employees working in different hospital sections and units (nurses and administrative personnel) were proportionally selected as the research sample.

The Persian version of Podsakoff׳s standard scale for measuring organizational citizenship behavior was used for data collection. It included two parts: one with 5 items relating to demographic information and the other with 24 items relating to organizational citizenship behavior (including 5 items for sportsmanship, 5 items for conscientiousness, 5 items for courtesy, 5 items for altruism, and 4 items for civic virtue). The items in the second part were scaled in Likert-type scale ranging from 1 (very low) to 5 (very high).

The validity and reliability of the Persian version of the scale have been confirmed in the study by Khalesi et al. [5] and the internal consistency of the scale amounted to *α* = 0.87 in our study. After obtaining the subjects׳ consent, they were asked to complete the scale by researcher׳s direct reference to the hospitals. The data were analyzed using SPSS 22and applying t-test, Tukey, and ANOVA.

### 2.1 Application to health-related policy-makings

There is not any study on Comparing the Organizational Citizenship Behavior in the Selected Hospitals with the Kind of Hospital Ownership as a Moderating Variable. findings of this study can be beneficial to hospital managers for improving performance and efficiency of health organization.
